# Improving patient-reported outcome measure scoring in shoulder trials: comparing classical test theory, item response theory, and adaptive testing approaches

**DOI:** 10.1302/2046-3758.1510.BJR-2026-0043.R1

**Published:** 2026-10-07

**Authors:** Jon P. Evans, Alex Mitchell, Jenna Shepherd, Harvinder Pal Singh, Amar Rangan

**Affiliations:** 1 Department of Public Health and Sports Science, University of Exeter, Exeter, UK; 2 Royal Devon and Exeter NHS Foundation Trust, Exeter, UK; 3 Department of Health Sciences, University of York, York, UK; 4 University Hospitals of Leicester NHS Trust, Leicester, UK; 5 University of Leicester School of Healthcare, Leicester, UK; 6 Nuffield Department of Orthopaedics, Rheumatology and Musculoskeletal Sciences, Botnar Research Centre, University of Oxford, Oxford, UK

**Keywords:** Patient-reported outcome measures, Shoulder surgery, Psychometrics, randomised controlled trial, patient-reported outcome measures (PROMs), shoulder, randomized controlled trials, Oxford Shoulder Score, statistical analysis, shoulder surgery, Frozen Shoulder, EQ-5D, surgical approaches, physiotherapy

## Abstract

**Aims:**

Patient-reported outcome measures (PROMs) underpin orthopaedic trials by capturing pain and function from the patient’s perspective. Traditionally, PROMs such as the Oxford Shoulder Score (OSS) are scored using classical test theory (CTT), which assumes equal item contribution and uniform measurement error. Item response theory (IRT) and computerized adaptive testing (CAT) offer modern alternatives that may improve precision and reduce respondent burden, but their impact on trial outcomes is unclear.

**Methods:**

We reanalyzed patient-level OSS data from two major UK randomized controlled trials, UK FROST (n = 503) and PROFHER (n = 250). We used three scoring approaches: CTT sum scores, IRT-based expected a posteriori (EAP) scores, and a simulated CAT. Original statistical analysis plans were replicated to compare treatment effect estimates, CIs, and p-values across methods. CAT simulations assessed potential reductions in questionnaire length.

**Results:**

Across both trials, treatment effect estimates and statistical significance were highly consistent between CTT, IRT, and CAT. Differences in magnitude were minimal, and overall trial conclusions were unchanged. Simulated CAT reduced median questionnaire length to four to five items at early follow-up, increasing to eight to nine items at later timepoints.

**Conclusion:**

In these large orthopaedic trials, modern psychometric methods produced results consistent with traditional scoring, but demonstrated potential efficiency gains. IRT and CAT may facilitate lower-burden, patient-centred outcome measurement and support innovative trial designs, without compromising the interpretation of treatment effects.

Cite this article: *Bone Joint Res* 2026;15(10):1206–1212.

## Article focus

- This study applies modern psychometric analysis methods (item response theory (IRT) and computerized adaptive testing (CAT)) to patient-reported outcome measures (PROMs) data from two major UK randomized controlled trials within shoulder surgery in order to evaluate these techniques compared to traditional approaches to analysis.

## Key messages

- Psychometric analysis methods produced results consistent with traditional approaches, with overall trial conclusions unchanged using these methods.
- IRT and CAT demonstrated efficiency gains and therefore may facilitate lower patient burden without compromising interpretation of treatment effects within trials.

## Strengths and limitations

- This study uses data from two major UK randomized controlled trials in shoulder surgery, replicating the original trial statistical analyses.
- This study furthers the exploratory use of IRT applications in classical test theory PROMs using established methodologies in large real-world datasets.
- The minimal clinically important difference (MCID) of IRT and CAT-based scores is uncertain, and future work is needed to develop robust IRT and CAT-based MCID for the Oxford Shoulder Score.

## Introduction

High-quality orthopaedic trials are critical to the delivery of evidence-based care. Increasingly, orthopaedic trials rely on patient-reported outcome measures (PROMs) to assess the effectiveness of interventions from the patient’s perspective.^1^ PROMs are designed to quantify symptoms, function, and quality of life, and have become essential for comparing the clinical benefit of surgical strategies or surgical versus non-surgical approaches.^2^

The Oxford Shoulder Score (OSS) is one of the most-used PROMs in shoulder trials.^3,4^ It has played a pivotal role in large, multicentre randomized controlled trials (RCTs) such as the UK FROST trial (comparing physiotherapy, injection, and surgery for frozen shoulder)^5^ and the PROFHER trial (evaluating surgery vs conservative treatment for proximal humeral fractures).^6^ Traditionally, PROMs like the OSS are scored using classical test theory (CTT), in which a patient’s score is the simple sum of their item responses.^7^ This approach is straightforward, but assumes that each item contributes equally to the total score and that measurement error is uniform across the scale. These assumptions may not hold in practice, and might, in theory, bias the results from clinical trials.^8^

Item response theory (IRT) provides a more sophisticated alternative. Rather than treating all items as equal, IRT models the relationship between each item and the underlying construct being measured (e.g. shoulder function or pain).^9^ Items are characterized by their difficulty (how severe a symptom must be for a respondent to endorse it) and discrimination (how well the item distinguishes between individuals with different levels of the underlying trait). IRT also allows for individual-level measurement error, offering more precise estimates of a patient’s status on a continuous scale.^10^

One common method of applying IRT is expected a posteriori (EAP) scoring, which integrates item characteristics and patient responses to generate a more nuanced and continuous estimate of the underlying construct. This method has been adopted in contemporary PROM frameworks such as PROMIS (Patient-Reported Outcomes Measurement Information System) and is increasingly recognized for its accuracy and interpretability.^11^ For example, two patients with the same sum score on a traditional scale may have different EAP scores if they endorsed different response patterns, which may lead to different clinical interpretations or research outcomes.

In addition to refining scoring, IRT enables the use of computerized adaptive testing (CAT). CAT dynamically selects items based on a patient’s previous responses, administering only the most informative questions for that individual. This reduces the burden on respondents while maintaining measurement precision.^12^ CAT approaches have already been validated for the OSS, demonstrating substantial reductions in questionnaire length with minimal loss of precision.^9^ By reducing respondent burden, alternative data collection frequencies may be possible, including ecological momentary assessment (EMA), where frequent, real-time data collection may minimize recall bias and provide insights into how the patient’s experience of their condition unfolds in everyday life.

Despite their growing use in psychometrics and health outcomes research, IRT and CAT methods have not been widely implemented in surgical trials. It remains unclear whether switching from traditional CTT sum-scoring to IRT-based scoring or CAT approaches would materially affect trial outcomes, such as treatment effect estimates or responder classifications. Harrison et al,^13^ in their reanalysis of The Total or Partial Knee Arthroplasty Trial (TOPKAT), found that psychometric sensitivity analysis, employing IRT methodologies, produced statistically significant interpretations in the trial outcome, although this difference did not reach clinically meaningful thresholds.

This study investigates the application of IRT-based scoring and CAT methodology to PROMs data from two major UK shoulder RCTs, UKFROST and PROFHER. We compare traditional CTT sum-scoring with IRT-based EAP scoring to determine whether the choice of scoring method influences trial conclusions. In addition, we explore whether CAT could feasibly be implemented using existing OSS items and whether this approach would lead to differences in efficiency and measured outcomes. By conducting these psychometric sensitivity analyses, we aim to assess the potential advantages of modern measurement techniques in orthopaedic trials and to inform the future design of PROM-based outcome evaluations.

## Methods

### Study context and data sources

This study used patient-reported outcome data from two major UK multicentre RCTs in shoulder surgery: the PROFHER (Proximal Fracture of the Humerus: Evaluation by randomization) trial and the UK FROST (UK Frozen Shoulder Trial) trial.

The UK FROST trial was a multicentre, pragmatic, three-arm, superiority randomized trial comparing three interventions for idiopathic frozen shoulder, early structured physiotherapy with steroid injection (ESP), manipulation under anaesthesia (MUA), and arthroscopic capsular release (ACR). A total of 503 participants (203 ESP; 201 MUA; 99 ACR) were randomized across 35 NHS centres, with OSS as the primary outcome at 12 months. Secondary outcomes were collected at three, six, and 12 months, and also included the EuroQol five-dimension five-level questionnaire (EQ-5D-5L)^14^ and range of motion assessments. Follow-up OSS data were available at all timepoints for 473 participants (191 ESP; 189 MUA; 93 ACR).^5^

The PROFHER trial was a pragmatic parallel-group, multicentre RCT comparing surgical versus non-surgical treatment in adults with displaced fractures of the proximal humerus. It recruited 250 patients (125 surgical; 125 non-surgical) across 32 UK centres, with follow-up assessments at six, 12, and 24 months. The primary outcome was the OSS at 24 months post-randomization, with additional assessments of function, quality of life, and complications. OSS data were available at six-, 12-, and 24-month timepoints for 232 (113 surgical; 119 non-surgical), 225 (111 surgical; 114 non-surgical), and 215 (106 surgical; 109 non-surgical) participants, respectively.^6^

### Oxford Shoulder Score and IRT calibration

The OSS is a 12-item PROM designed to measure shoulder pain and function. Each item is rated on a five-point Likert scale, producing a total score range from 0 (worst) to 48 (best function). In a prior psychometric evaluation, we confirmed that the OSS meets key assumptions of IRT, including unidimensionality (all items measure a single underlying construct, such as shoulder function), monotonicity (patients with better function are more likely to endorse better response options), and local independence (once shoulder function is accounted for, item responses are statistically independent of each other). Using a graded response model (GRM), item-level difficulty and discrimination parameters were established and validated in a large NHS registry dataset.^7^

### Analysis strategy

To evaluate the impact of different scoring approaches, we reanalyzed outcome data from both trials using the original trial datasets and statistical analysis plans.

For the CTT analysis, we began by replicating the original trial analyses using traditional CTT-based OSS sum scores. This included applying the original statistical analysis plans for each trial, using the same outcome timepoints and thresholds for statistical significance. In both UK FROST and PROFHER, the OSS was analyzed following the intention-to-treat principle and using a mixed-effects repeated measures model incorporating all timepoints with an unstructured covariance pattern. In UK FROST, the model included treatment group, timepoint, treatment × time interaction, age (in years), sex (male or female), diabetes status (diabetic or non-diabetic), and baseline OSS as fixed effects. In PROFHER, the model included treatment group, timepoint, treatment × time interaction, tuberosity involvement at baseline (yes or no), age (< 65 years or ≥ 65 years), sex (male or female), and baseline EQ-5D score. The model used in UK FROST included participant and recruiting site as random effects, while the model used in PROFHER included participant as a random effect. p-values in the original UK FROST analysis were derived using two-tailed Wald tests.

Next, we applied IRT-based scoring to the same patient-level OSS data using the previously derived GRM item parameters.^9^ Each participant’s latent trait estimate was calculated using the EAP method using the mirt package in R (v. 1.44.0, R Foundation for Statistical Computing, Austria). This produces scores on a logit scale with a range of approximately −4 to 4. Transformation of this scale to mirror the 0 to 48 scale of the original OSS was not undertaken. The trial analyses were then repeated using these IRT-derived scores, mirroring the original analytic strategy and maintaining consistency with the pre-specified p-values.

Finally, we evaluated the feasibility and impact of implementing CAT using the OSS in these trials. Simulated CAT administrations were conducted using the Firestar package in R (v. 1.3.2), applying a standard error stopping rule of 0.32 (equivalent to a reliability of 0.90), with a minimum number of items set to 2, maximum of 12 (full-scale) using the trial participant data. For each simulated participant, we also recorded the number of items required. The resulting IRT-CAT scores from the CAT simulation were then used to repeat the original statistical analysis plan.

### Statistical analysis

Analyses were conducted using R (R Foundation for Statistical Computing) and Stata v. 18.0 (StataCorp, USA). The statistical workflow was first developed and tested on synthetic data to ensure the validity of the pipeline. Once validated, it was applied to the original trial datasets. Trial outcome comparisons were assessed using the same statistical thresholds as reported in the original publications. A 5% statistical significance level was used in all analyses.

## Results

The full results of the PROFHER and UK FROST trials have been previously published.^5,6^

### Trial sensitivity analysis

For UK FROST, estimates of the mean difference in OSS between treatment groups calculated using CTT, IRT, and CAT scoring approaches are reported (with 95% CIs and p-values) at three, six, and 12 months, as well as averaged over all timepoints (Table I).

**Table I.** Comparison of OSS scores in the UK FROST Trial using CTT to IRT to and CAT scoring methods for treatment groups (MUA to ESP, ACR) across timepoints.

Scoring method	MUA vs ESP		ACR vs ESP		ACR vs MUA	
CTT	MD (95% CI)	p-value	MD (95% CI)	p-value	MD (95% CI)	p-value
Averaged over 12 months	0.61 (−1.31 to 2.53)	0.53	−0.23 (−2.15 to 1.70)	0.82	−0.84 (−2.41 to 0.72)	0.29
Month 3	−1.36 (−3.70 to 0.98)	0.25	−4.72 (−7.06 to −2.39)	< 0.01	−3.36 (−5.27 to −1.45)	0.06
Month 6	2.15 (−0.12 to 4.42)	0.06	0.98 (−1.31 to 3.26)	0.40	−1.17 (−3.02 to 0.67)	0.21
Month 12	1.05 (−1.28 to 3.39)	0.38	3.06 (0.71 to 5.41)	0.01	2.01 (0.10 to 3.91)	0.04
**IRT**						
Averaged over 12 months	0.11 (−0.12 to 0.33)	0.36	−0.01 (−0.24 to 0.22)	0.93	−0.12 (−0.30 to 0.07)	0.22
Month 3	−0.09 (−0.35 to 0.16)	0.47	−0.47 (−0.72 to −0.21)	< 0.01	−0.37 (−0.58 to −0.16)	< 0.01
Month 6	0.24 (−0.03 to 0.51)	0.08	0.03 (−0.24 to 0.30)	0.82	−0.21 (−0.43 to 0.01)	0.06
Month 12	0.17 (−0.12 to 0.46)	0.26	0.40 (0.11 to 0.70)	0.01	0.24 (0 to 0.47)	0.05
**CAT**						
Averaged over 12 months	0.12 (−0.09 to 0.32)	0.26	0.02 (−0.19 to 0.22)	0.88	−0.10 (−0.27 to 0.07)	0.24
Month 3	−0.06 (−0.28 to 0.17)	0.63	−0.36 (−0.58 to −0.14)	< 0.01	−0.31 (−0.49 to −0.12)	< 0.01
Month 6	0.23 (−0.02 to 0.48)	0.08	0.04 (−0.21 to 0.29)	0.76	−0.19 (−0.39 to 0.01)	0.07
Month 12	0.18 (−0.09 to 0.45)	0.20	0.37 (0.10 to 0.64)	0.01	0.19 (−0.03 to 0.41)	0.09

ACR, arthroscopic capsular release; CAT, computerized adaptive testing; CTT, classical test theory; ESP, early structured physiotherapy with steroid injection; IRT, item response theory; MD, mean difference; MUA, manipulation under anaesthesia; OSS, Oxford Shoulder Score.

Across all timepoints and treatment comparisons (MUA vs ESP; ACR vs ESP; and ACR vs MUA), the magnitude and direction of treatment effects were highly consistent across scoring methods. p-values and 95% CIs showed substantial overlap between CTT, IRT, and CAT approaches.

At three months, both the ACR vs ESP and ACR vs MUA comparisons demonstrated statistically significant differences across all methods. The CAT-derived estimates tended to be slightly smaller in absolute magnitude compared with IRT, although this did not affect overall interpretation.

At six months, no comparisons reached statistical significance in any method. Mean differences across all approaches remained small and aligned in direction and scale.

At 12 months, the ACR versus ESP comparison reached statistical significance across all methods. The ACR versus MUA comparison was statistically significant using CTT and IRT, and borderline using CAT (p = 0.09).

When treatment effects were averaged (mean) over the 12-month follow-up period, no comparisons were statistically significant, and all methods produced closely aligned results.

For PROFHER, estimates of the mean difference in OSS between the surgical and non-surgical treatment groups calculated using CTT, IRT, and CAT scoring approaches are reported (with 95% CIs and p-values) at six, 12, and 24 months, as well as averaged (mean) across the full 24-month follow-up period (Table II).

**Table II.** Comparison of OSS scores in the PROFHER trial using CTT, IRT, and CAT scoring methods between surgery and non-surgery groups over a two-year follow-up period.

Time period	CTT scoring surgery vs non-surgery		IRT scoring surgery vs non-surgery		CAT scoring surgery vs non-surgery	
	MD (95% CI)	p-value	MD (95% CI)	p-value	MD (95% CI)	p-value
Averaged over 2 years	0.75 (-1.33 to 2.84)	0.48	0.09 (-0.12 to 0.30)	0.42	0.13 (-0.08 to 0.38)	0.22
Month 6	2.25 (-0.07 to 4.57)	0.06	0.24 (0 to 0.52)	0.05	0.25 (0.01 to 0.55)	0.04
Month 12	0.42 (-1.78 to 2.63)	0.71	0.11 (-0.11 to 0.33)	0.31	0.21 (-0.02 to 0.47)	0.08
Month 24	-0.29 (-2.53 to 1.95)	0.80	-0.07 (-0.30 to 0.16)	0.56	-0.03 (-0.26 to 0.23)	0.79

CAT, computerized adaptive testing; CTT, classical test theory; IRT, item response theory; MD, mean difference; OSS, Oxford Shoulder Score.

Across all timepoints, the magnitude and direction of treatment effects were broadly consistent across scoring approaches. CIs and p-values were closely aligned between CTT, IRT, and CAT methods, suggesting a high degree of agreement between scoring techniques.

At six months, treatment effects were largest and approached statistical significance using CTT (p = 0.06), and reached significance using IRT (p = 0.05) and CAT (p = 0.04). Although the absolute values of the mean differences differed slightly across methods, all three approaches indicated a small-to-moderate benefit of surgery at this early follow-up point.

At 12 months, treatment effects were reduced in magnitude and no longer statistically significant in any scoring method. Mean differences remained directionally consistent with earlier findings, with slightly attenuated CAT-based estimates compared to IRT.

At 24 months, treatment effects were minimal and non-significant across all methods. The mean differences were close to zero, and CIs spanned both positive and negative values, indicating no evidence of sustained benefit from surgery over non-surgical management at this timepoint.

When averaged over the two-year follow-up period, no scoring method demonstrated a statistically significant difference between surgical and non-surgical groups. All three approaches yielded small, non-significant mean differences, with overlapping confidence intervals and consistent directionality.

Together, these findings suggest that the choice of scoring methodology (CTT, IRT, or CAT) had minimal impact on the interpretation of treatment effects in the PROFHER trial.

### Computerized adaptive test analysis

The CAT algorithm reduced the number of OSS items required to estimate participant scores across both trials. However, the number of items administered varied by timepoint, generally increasing later in follow-up (Table III).

**Table III.** Number of items completed at each assessment timepoint in the UK FROST and PROFHER trials.

Trial	Timepoint	Median items (IQR)	Absolute range
UK FROST	Baseline	4 (3 to 5)	2 to 12
	3 mths	4 (3 to 6)	2 to 12
	6 mths	5 (3 to 11)	2 to 12
	12 mths	8 (4 to 12)	2 to 12
PROFHER	6 mths	5 (3 to 11)	2 to 12
	12 mths	8 (4 to 12)	2 to 12
	24 mths	9 (5 to 12)	2 to 12

The number of OSS items administered at each follow-up timepoint in the UK FROST Trial can be found in Table III and Figure 1. These results demonstrate that most participants required fewer than the full 12-item questionnaire. However, item usage increased at later timepoints, likely due to greater variability in recovery and a need for additional items to achieve the required standard error threshold in participants at the extremes of shoulder function.

**Fig. 1:**
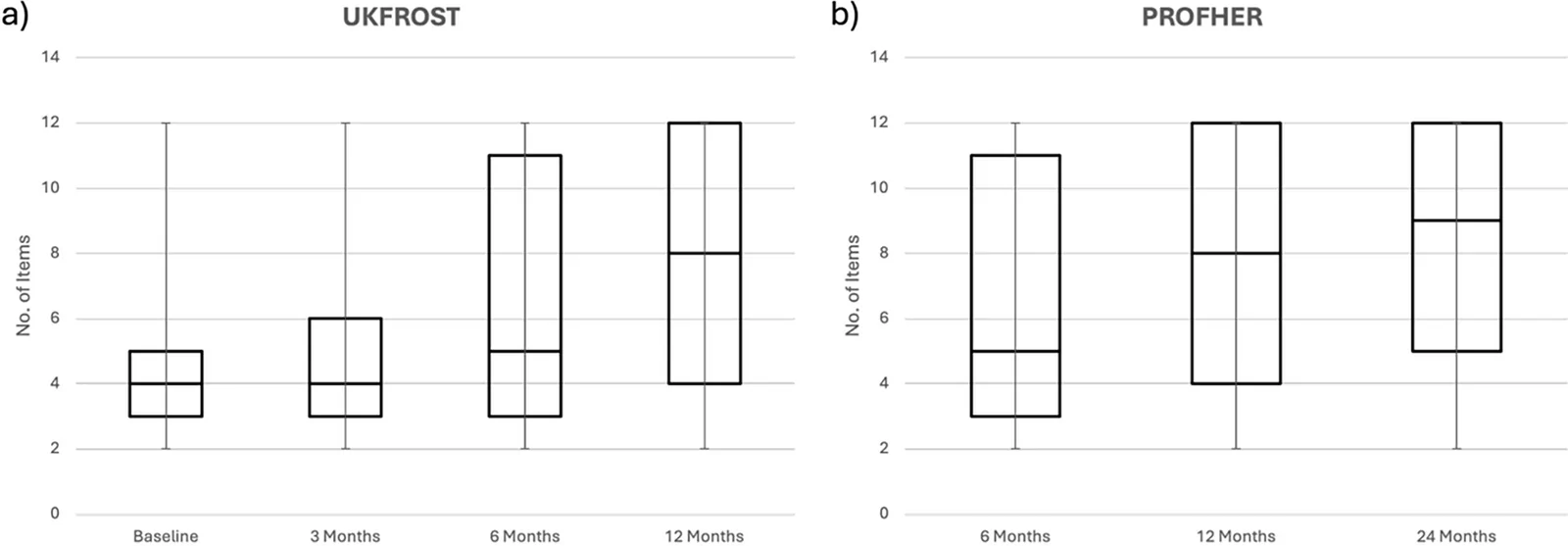
Box and whisker plot presenting median, IQR, and absolute range of number of items completed at each assessment timepoint in the a) UK FROST and b) PROFHER trials.

The pattern of item usage was broadly similar in the PROFHER trial, the results of which can be found in Table III and Figure 1. Again, the number of items increased with time since randomization. The widest range was observed at 24 months, reflecting heterogeneity in long-term outcomes and variability in participant response patterns.

Across both UK FROST and PROFHER, CAT substantially reduced respondent burden, particularly in early follow-up. However, more items were required at later timepoints to maintain precision in scoring, consistent with greater dispersion in pain and functional outcomes and limitations in information coverage at the extremes of the latent trait.

## Discussion

This study compared three approaches to scoring the OSS in two large orthopaedic trials, traditional CTT, IRT, and CAT. Following scoring, the trials’ original statistical analyses were replicated, and across all methods treatment effect estimates and p-values were substantively similar. This suggests that, at the group level, the scoring method may have limited influence on primary outcome interpretation in pragmatic orthopaedic trials when a well-designed, psychometrically robust PROM is used. However, comparisons between these approaches have highlighted that measurement efficiency, trial design, and the evolving potential of adaptive methods may be leveraged when modern psychometric scoring is applied.

The apparent equivalence across methods likely reflects the strong construct validity of the OSS, which has been shown to be a reliable and responsive instrument in shoulder conditions.^15^ That both IRT and CAT methods converged with traditional scoring supports the robustness of this instrument across different psychometric frameworks. Nonetheless, equivalence at the level of overall scores should not obscure the potential for more granular differences. Future work may evaluate measurement invariance and differential item functioning (DIF), particularly across subgroups defined by age, sex, surgical intervention, or comorbidities. DIF occurs when individuals from different groups, but with the same underlying level of the latent trait being measured (e.g. shoulder pain or function), respond differently to PROM questions.^16^ While we did not undertake DIF analyses here, it may identify whether specific items behave differently in different populations, and whether IRT offers advantages in maintaining fairness and validity in subgroup comparisons.

Despite similar point estimates, IRT-based scoring has important practical and statistical advantages, particularly in secondary analysis or prospective trial planning. Because IRT models individual item characteristics, they can accommodate missing data more flexibly than CTT, providing valid person scores even when some items are missing, which may be an important consideration in trials with incomplete PROM returns. This extends the utility of partially completed forms and supports better use of real-world data and trial efficiency. In contrast, CTT approaches often require full completion of all items, or poorly scaled methods to deal with data loss which leads to unnecessary data attrition and reduced power.

While treatment effects were consistent across all scoring methods, CIs were generally narrower with CAT compared to IRT, suggesting improved measurement precision. This may allow for reduced sample size requirements in future trials, particularly in musculoskeletal research where recruitment can be challenging and PROMs often serve as primary endpoints. The attenuation of mean differences observed may reflect reduced score variability and greater precision, especially where item banks are most informative at mid-range functional levels. These findings highlight a potential trade-off between measurement efficiency and sensitivity to change, which should be considered when implementing CAT in heterogeneous clinical populations. Although the direction and relative magnitude of treatment effects were consistent across methods, absolute values differ due to the underlying scoring scales, CTT produces unstandardized scores (OSS 0 to 48), while IRT and CAT operate on standardized latent trait metrics. As such, while direct comparison of CIs between CTT and IRT/CAT is inappropriate, the consistent patterns of statistical significance across methods support the robustness of findings under different psychometric scoring approaches. While rescaling IRT/CAT scores to the CTT metric (e.g. 0 to 48 OSS) allows side-by-side comparison, it can obscure the psychometric advantages of IRT, misrepresent measurement error, and introduce misleading assumptions about equivalence between fundamentally different scoring models.

Our findings are in line with previous work applying CAT methods,^9^ suggesting the potential for reduced respondent burden, with potential applications across trials and registries collecting PROMs. Our analysis of the CAT algorithm revealed meaningful trends in item burden over time. At earlier timepoints, such as baseline and three months, fewer OSS items were required to estimate scores to a high level of predefined precision. As follow-up progressed, the number of items increased, particularly at 12 and 24 months. This may represent the wider spread of patient outcomes at later stages of recovery and the reduced information available from each item at the extremes of shoulder function, where the OSS has previously been shown to be limited.^17^ When participants approach full recovery or report very low function, more items are needed to resolve score estimates to a consistent level of standard error. An additional explanation for the increased item requirement at later follow-up may relate to the multidimensional nature of the OSS. Although modelled as unidimensional, the instrument captures both pain and function, which may recover at different rates following intervention. Divergence between these domains may reduce the precision of a single latent trait estimate, requiring additional items for the CAT algorithm to resolve the underlying construct. This effect may be accentuated in heterogeneous trial populations with variable recovery trajectories.

The implication is that CAT may be most efficient when measuring moderate levels of disability or in early recovery phases. At longer-term follow-up points, or when greater precision is required at the tails of the distribution, the efficiency of CAT may diminish, particularly if the item bank does not sufficiently cover the full range of the latent trait. This suggests a potential role for expanded item banks that encompass a broader range trait levels,^18^ or longitudinally recalibrated item banks, especially for studies seeking to optimize PROM efficiency across diverse recovery trajectories.

From an implementation perspective, several practical challenges must be considered before CAT can be routinely adopted in clinical trials. These include the need for appropriate digital infrastructure, participant familiarity with electronic questionnaires, and regulatory clarity on how adaptive instruments can be monitored and reported.

Moreover, the adaptive nature of CAT opens new opportunities for high-frequency, low-burden data collection, such as through EMA^19^ or app-based PROM follow-up. In this context, CAT could support more responsive, participant-centred trials by reducing recall bias and enabling real-time monitoring of recovery trajectories.^20^ Such approaches may also facilitate adaptive trial designs, where treatment pathways can be altered dynamically in response to PROM trends but, as demonstrated here, still utilizing well-accepted PROMs.

Several limitations of this work must be acknowledged. First, this was a secondary data analysis from two trials not originally designed to test IRT or CAT methods. While we preserved the original analysis plan and endpoints, this work was exploratory and not powered to detect subtle differences between scoring approaches. Second, we used the Firestar simulation platform to estimate CAT item usage retrospectively; this does not fully replicate real-time CAT administration, which may be influenced by respondent burden, item exposure, or stopping rule optimization. Third, although IRT models permit sophisticated treatment of missing data, the presence and pattern of missingness may still bias estimation in ways we did not explore here. Fourth, we did not evaluate the psychometric behaviour of the OSS over time, and our use of a single static item calibration may not fully capture potential changes in item performance across recovery phases.

An important further limitation of our analysis is that we did not derive new minimal clinically important difference (MCID) thresholds for IRT or CAT-based scores. While group-level effects were consistent, estimating MCIDs within an IRT framework requires anchor-based methods that are specifically calibrated to the latent trait continuum.^21^ Applying existing CTT-derived MCIDs to IRT scores may be misleading, and risks under- or over-interpreting meaningful changes. Future work is needed to develop robust, empirically derived IRT-based MCIDs for the OSS.

In conclusion, this study supports the use of IRT and CAT scoring in orthopaedic trials and suggests that the OSS performs well across scoring paradigms. While the overall interpretation of trial results was consistent across methods, IRT offers important methodological flexibility, and CAT presents an opportunity to reduce respondent burden. These methods may enable more efficient and patient-centred outcome measurement, especially if linked to expanded item banks, real-time administration, and responsive trial designs. Further work is needed to optimize these tools for use in routine orthopaedic research and clinical practice.

## Data Availability

The datasets generated and analyzed in the current study are not publicly available due to data protection regulations. Access to data is limited to the researchers who have obtained permission for data processing. Further inquiries can be made to the corresponding author.
